# Systems metabolic insights into kaempferol-mediated alleviation of alcoholic fatty liver disease

**DOI:** 10.3389/fendo.2025.1610613

**Published:** 2025-05-29

**Authors:** Chenmeng Song, Lijia Li, Jia Li, Jengyuan Yao

**Affiliations:** ^1^ School of Public Health, Fujian Medical University, Fuzhou, China; ^2^ Key Laboratory of Functional And Clinical Translational Medicine, Xiamen Medical College, Fujian Province University, Xiamen, China

**Keywords:** metabolomics, alcoholic fatty liver disease (AFLD), kaempferol, oxidative stress, lipid metabolism

## Abstract

**Introduction:**

Alcoholic fatty liver disease (AFLD) is a significant global health concern caused by chronic and excessive alcohol consumption, leading to hepatic steatosis, inflammation, and metabolic disruption. Currently, there are limited effective therapeutic options. Kaempferol, a dietary flavonoid, has shown potential for regulating oxidative stress and lipid metabolism.

**Methods:**

In this study, C57BL/6 mice (n = 12 per group) were assigned to control, ethanol-fed (AFLD), or ethanol plus kaempferol (KPF) groups. The AFLD model was induced using a modified Lieber-DeCarli diet with stepwise ethanol escalation and a final single binge dose. Kaempferol was administered at 20 mg/kg/day by gavage. Serum biochemical markers, liver histology, oxidative stress indices, and untargeted plasma metabolomics were assessed to evaluate KPF’s protective effects.

**Results:**

Kaempferol-treated mice exhibited significantly reduced alanine aminotransferase (ALT, p < 0.0001) and aspartate aminotransferase (AST, p < 0.05) levels compared to the AFLD group. Histological analysis revealed improved hepatic architecture and reduced lipid droplet accumulation. Kaempferol also enhanced antioxidant capacity, evidenced by reduced malondialdehyde (MDA, p < 0.05). Metabolomic profiling identified multiple altered metabolites associated with glycolysis, amino acid metabolism, and sphingolipid signaling.

**Discussion:**

These findings indicate that kaempferol exerts protective effects against AFLD through multi-target regulation of oxidative stress and metabolic pathways. Our study provides systems-level evidence supporting kaempferol as a promising dietary-based intervention for AFLD management.

## Introduction

Alcohol consumption is a major contributor to various health conditions, including alcoholic fatty liver disease (AFLD), which poses significant health and socioeconomic challenges. AFLD results from excessive alcohol intake, leading to the production of toxic intermediates such as acetaldehyde and reactive oxygen species (ROS) in the liver (1). These intermediates induce oxidative stress, mitochondrial damage, and lipid peroxidation (2, 3), ultimately resulting in hepatocyte injury, inflammation, fibrosis, and progressive liver damage (4).

The liver is central to lipid metabolism, processing free fatty acids from dietary intake, extrahepatic sources, and glycolysis. These fatty acids undergo β-oxidation, membrane synthesis, or esterification into triglycerides, which are subsequently packaged into very-low-density lipoproteins (VLDL) for secretion. Chronic alcohol consumption disrupts these pathways, impairing lipid metabolism and leading to lipid accumulation, a hallmark of fatty liver (5). Specifically, ethanol metabolism increases the hepatic NADH/NAD^+^ ratio, which inhibits mitochondrial β-oxidation of fatty acids. Simultaneously, alcohol activates lipogenic transcription factors such as SREBP-1c, enhancing *de novo* lipogenesis. These alterations contribute to the accumulation of triglycerides within hepatocytes, forming lipid droplets and leading to hepatic steatosis (6).

Genetic predisposition may further influence susceptibility to AFLD, but large-scale studies validating these associations are limited (7). Given the multifaceted pathophysiology of AFLD, effective prevention strategies must extend beyond reducing alcohol consumption to include targeted interventions addressing its underlying mechanisms.

Kaempferol (KPF) is a dietary flavonoid widely distributed in vegetables, fruits, and traditional medicinal plants, and it exhibits potent antioxidant, anti-inflammatory, and metabolic regulatory activities in various preclinical models (8–11). For instance, *in vitro* and *in vivo* research indicates that KPF can modulate pivotal signaling pathways (e.g., Nrf2/HO-1, PI3K/AKT, and NF-κB) to alleviate oxidative stress, inflammation, and lipid dysregulation, thereby offering therapeutic benefits in metabolic diseases such as diabetes, obesity, and non-alcoholic fatty liver disease (NAFLD) (8, 9). Evidence from rodent models of myocardial ischemia–reperfusion injury and adenine-induced chronic kidney disease, as well as from H9c2 cardiomyocytes and HK-2 renal tubular cell cultures, shows that kaempferol attenuates oxidative damage, down-regulates pro-fibrotic signaling, and preserves tissue architecture in both cardiovascular and renal settings (10, 11). Although these findings support KPF as a multi-target agent against metabolic disturbances, additional clinical research is needed to fully ascertain its safety and efficacy.

This study aims to evaluate the protective effects of KPF on alcohol-induced hepatic steatosis and injury, focusing on its ability to regulate oxidative stress and lipid metabolism. Using C57BL/6 mice subjected to a high-dose, periodic ethanol-intake regimen to mimic acute alcohol-induced hepatic injury, we investigated KPF’s efficacy by assessing liver histopathology, functional biomarkers (ALT, AST), lipid metabolism parameters (TC, TG, LDL-C, HDL-C), and oxidative stress indicators (MDA, SOD, GSH-Px). Three groups were included: a control group (CTRL, standard liquid diet without ethanol), an ethanol-fed group (AFLD), and an ethanol-fed group receiving kaempferol supplementation (KPF). These findings provide mechanistic insights and a foundation for developing KPF as a therapeutic agent for AFLD, leveraging its antioxidant and metabolic regulatory properties.

## Materials and methods

### Materials

Kaempferol (KPF) (catalog no. K812226) was purchased from Macklin (Shanghai, China). Assay kits for alanine aminotransferase (ALT; catalog no. C009-1-1), aspartate aminotransferase (AST; catalog no. C010-1-1), triglyceride (TG; catalog no. A110-1-1), total cholesterol (TC; catalog no. A111-1-1), high-density lipoprotein cholesterol (HDL-C; catalog no. A112-1-1), low-density lipoprotein cholesterol (LDL-C; catalog no. A113-1-1), malondialdehyde (MDA; catalog no. A003-1-2), superoxide dismutase (SOD; catalog no. A001-3-2), and glutathione peroxidase (GSH-Px; catalog no. A005-1-2) were obtained from Nanjing Jiancheng Bioengineering Institute (Nanjing, China). The Oil Red O staining kit was sourced from Beyotime (Shanghai, China). Primary antibodies included anti-HO-1 (ab305290, Abcam, USA), anti-Nrf2 (SC-365949, Santa Cruz, USA), and HRP-conjugated β-Actin (HRP-66009, Proteintech, Wuhan, China). The secondary antibody, HRP-conjugated Goat anti-rabbit IgG, was purchased from Abcam (ab288151).

### Animal experiments

Male C57BL/6 mice (6–8 weeks old, specific pathogen-free) were purchased from Shanghai Slack Laboratory Animal Co., Ltd. (Production License SCXK(HU)2022-0004) and housed under controlled conditions (temperature 18–26°C, relative humidity 40–60%), with a normal light-dark cycle and free access to food and water. To adapt the animals, all mice were first given a Lieber-DeCarli-based liquid diet for one week, during which the last three days involved a standard liquid diet without ethanol. These mice were chosen because the C57BL/6 strain reliably develops pronounced steatosis, oxidative damage, and inflammatory responses under Lieber-DeCarli or NIAAA chronic-plus-binge ethanol feeding, making it a standard model for experimental AFLD (5, 12).

After adaptation, the mice were randomly divided into three groups (n=12 per group). The CTRL group was maintained on the standard liquid diet and received daily vehicle gavage. The AFLD group received an ethanol-containing liquid diet plus vehicle gavage. The KPF group received the same ethanol-containing liquid diet supplemented with kaempferol solution (4 mg/mL, 0.1 mL/20 g body weight) by gavage. This dosage was selected based on prior studies demonstrating hepatoprotective effects against alcoholic liver injury in mice without evident toxicity (8, 12), and was further validated through our own preliminary experiments confirming biological efficacy and safety at this dose. The Lieber-DeCarli liquid diet was prepared according to the manufacturer’s instructions (Dyets, USA). For example, the 1% (v/v) ethanol formula per 1 kg diet contained 132.18 g L10016A base powder, 71.68 g maltodextrin, 10 g anhydrous ethanol, and 786.14 g deionized water. Diets with 2%, 3%, and 4% ethanol were produced by proportionally increasing the ethanol volume (20, 30, and 40 g, respectively) while reducing water to maintain equal total volume and identical nutrient composition. During the feeding period, ethanol concentration in the liquid diet was gradually increased, starting at 1% on Day 1, 2% on Day 3, 3% on Day 4, and 4% from Day 6 to Day 15. On Day 16, to simulate binge drinking (13), the AFLD and KPF groups received a single high dose of ethanol (5 g/kg, 31.5% v/v) by gavage, while the CTRL group received an equal volume of maltodextrin. Twenty-four hours later, the mice were fasted, anesthetized with tribromoethanol, and blood was collected via cardiac puncture. Plasma and liver samples were harvested and stored at −80°C for further analyses.

All procedures were reviewed and approved by the Experimental Animal Ethics Committee of Xiamen Medical College (Approval Number 20240207015, February 7, 2024) and were conducted in compliance with the ARRIVE guidelines to ensure animal welfare.

### Biochemical analysis

Plasma levels of ALT and AST were measured to evaluate liver injury. Lipid metabolism was assessed by quantifying plasma levels of TG, TC, HDL-C, and LDL-C. Oxidative stress markers, including MDA, SOD, and GSH-Px, were analyzed in liver tissues following manufacturer protocols.

### Liver histological analysis

Liver tissues were fixed in 4% paraformaldehyde overnight, embedded in paraffin, and sectioned into 4 μm slices for hematoxylin and eosin (H&E) staining. Frozen liver tissues were sectioned into 8 μm slices and stained with Oil Red O to evaluate lipid accumulation. Histological imaging was performed using an optical microscope (Leica, Wetzlar, Germany). All slides were coded and independently evaluated under double-blind conditions by two board-certified pathologists. The locations of hepatocyte hypertrophy and lipid vacuolation were qualitatively identified and annotated by both pathologists. For Oil Red O staining, lipid-positive areas (%) were quantified from three randomly selected 400× fields per section using ImageJ (NIH, USA), and the average values were used for statistical analysis.

### Western blot analysis

Liver tissues were homogenized in RIPA buffer containing protease inhibitors, followed by ultrasonic disruption. After centrifugation at 12,000 rpm for 10 minutes, the supernatant protein concentrations were determined using a BCA protein assay kit. Equal amounts of protein were resolved via SDS-PAGE, transferred to PVDF membranes, and blocked with 5% skim milk for 1 hour. Membranes were incubated overnight at 4°C with specific primary antibodies, followed by secondary antibody incubation at room temperature for 1 hour. Signals were detected using a chemiluminescence imaging system, and band intensities were quantified using ImageJ software (v1.54g).

### Metabolomics sample preparation

Plasma samples were thawed on ice to preserve integrity. For each sample, 50 μL of plasma was mixed with 200 μL of methanol, followed by ultrasonic mixing to ensure uniformity. The mixture was incubated at 4°C for 1 hour to precipitate proteins and centrifuged at 15,000 g for 10 minutes at 4°C. The supernatants were carefully collected, dried under nitrogen, and resuspended in 100 μL of acetonitrile-water solution (1:1, v/v) for HPLC-MS/MS analysis.

### Metabolomics analysis

Metabolomic profiling was conducted using a Waters Xevo G2-XS QToF mass spectrometer coupled with an ACQUITY UPLC BEH C18 column (2.1 mm × 50 mm, 1.8 μm; Waters Corp., Milford, USA). Chromatographic separation was performed at 20°C using a Waters Acquity™ UPLC system. The mobile phase consisted of formic acid aqueous solution (solvent A) and acetonitrile (solvent B) at a flow rate of 0.4 mL/min, with a gradient program of 0–1 min: 10% B; 1-7.5 min: 10% → 65% B; 7.5-10.5 min: 65% → 100% B; and 10.5–12 min: 100% → 10% B. The injection volume was 2 μL.

The mass spectrometer operated in both positive and negative ionization modes, with a scan range of 50–1200 Da. Key parameters included a cone voltage of 40 kV, a capillary voltage of 3.0 kV, a desolvation gas flow rate of 800 L/h at 450°C, and a source temperature of 100°C. Mass calibration was performed using leucine enkephalin, with [M+H]+ at m/z 556.2771 in positive mode and [M-H]− at m/z 554.2615 in negative mode. Data acquisition was performed using MassLynx™ V4.1 software, and preprocessing, including peak alignment and normalization, was conducted with Progenesis QI V2.1 software (Waters Corp., Milford, MA, USA).

Multivariate statistical analyses, including principal component analysis (PCA) and orthogonal partial least squares discriminant analysis (OPLS-DA), were conducted using Origin software (Origin Lab, Northampton, USA). Metabolites were selected based on Variable Importance in Projection (VIP) > 1, fold change (FC) > 1.5, and p < 0.05. Metabolite annotation was performed using KEGG (https://www.kegg.jp/) and HMDB (http://www.hmdb.ca/) databases, with pathway enrichment analysis conducted through MetaboAnalyst 6.0 (https://www.metaboanalyst.ca/). Differential metabolites with AUC > 0.8 were further analyzed as potential biomarkers.

### Statistical analysis

Data were expressed as mean ± SEM (or SD, as specified). Statistical comparisons were performed using One-way ANOVA, and if an overall difference was found, Tukey’s *post-hoc* analysis was applied. Significance was set at p < 0.05. All statistical tests were conducted in GraphPad Prism 9.

## Results

### KPF alleviates alcohol-induced liver injury and oxidative stress

**Figure 1:**
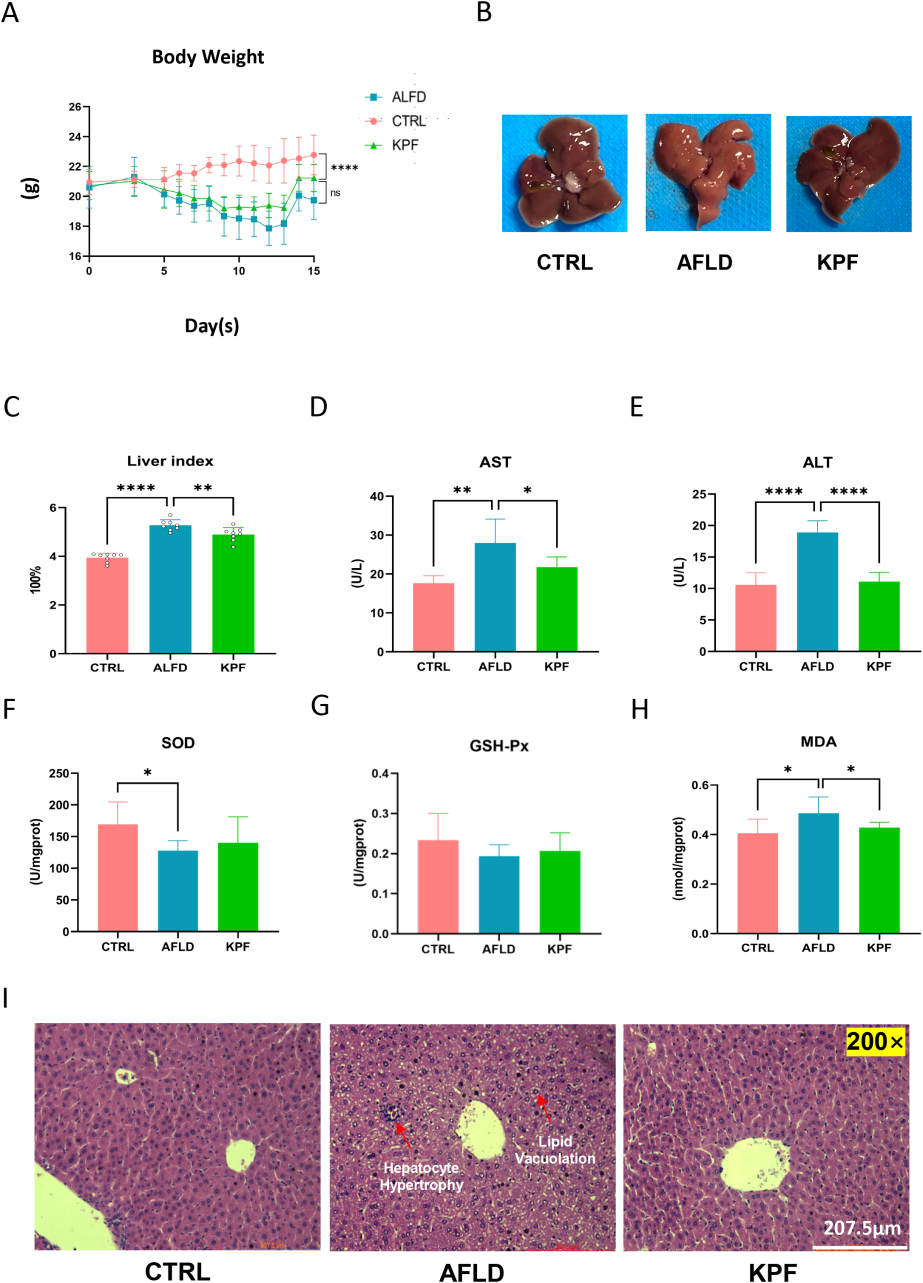
KPF Alleviates Alcohol-Induced Liver Injury and Oxidative Stress **(A)** Body weight changes of mice during the feeding period with an alcohol-containing diet. **(B)** Morphological images of the liver, demonstrating that KPF treatment reduced liver steatosis compared to the AFLD group. **(C)** Liver index in the three groups. **(D)** Plasma AST levels. **(E)** Plasma ALT levels. **(F)** SOD levels in liver tissue. **(G)** GSH-Px levels in liver tissue. **(H)** MDA levels in liver tissue. **(I)** Representative H&E-stained liver sections depicting liver histology (magnification 200×). KPF treatment alleviated alcohol-induced pathological changes. All data were analyzed by one-way ANOVA followed by Tukey’s *post-hoc* test; *p < 0.05, **p < 0.01, ****p < 0.0001, ns, not significant (n = 12 per group).

In the CTRL group, body weight steadily increased throughout the experimental period, whereas the AFLD and KPF groups, which were fed an alcohol-containing liquid diet, initially experienced weight loss followed by a slight recovery. Notably, the weight gain in the AFLD group was significantly lower than that in the KPF group (**Figure 1A**). Observation of liver appearance revealed reduced steatosis in the KPF group, evidenced by darker coloration compared to the AFLD group, which exhibited the lightest liver color (**Figure 1B**). The liver index rose from 3.94 ± 0.18% in CTRL mice to 5.28 ± 0.23% in the AFLD group (p < 0.0001 vs. CTRL); kaempferol treatment partially reversed this increase, reducing the index to 4.89 ± 0.28% (p < 0.01 vs. AFLD), as shown in **Figure 1C**.

Plasma levels of ALT and AST, key indicators of liver injury, were significantly elevated in the AFLD group compared to the CTRL group. KPF treatment significantly decreased these levels, with AST showing notable improvement (p < 0.05) and ALT showing a pronounced reduction (p < 0.0001) (**Figures 1D, E**). Antioxidant marker analysis in liver tissues revealed that SOD activity, which was significantly reduced in the AFLD group (p < 0.05), increased in the KPF group, although this increase did not reach statistical significance. Similarly, GSH levels were partially restored in the KPF group but remained statistically insignificant. In contrast, MDA levels, a marker of oxidative stress, were significantly elevated in the AFLD group (p < 0.05) and were significantly reduced following KPF treatment (p < 0.05) (**Figures 1F–H**).

Histopathological examination demonstrated normal liver morphology in the CTRL group, characterized by well-organized hepatocytes with no vacuolar degeneration, inflammation, or necrosis. In contrast, the AFLD group exhibited disorganized hepatic architecture, hepatocyte hypertrophy, and extensive lipid vacuolation in the cytoplasm. These pathological changes were notably reversed in the KPF-treated group, with restoration of liver structure and a reduction in lipid vacuolation (**Figure 1I**).

### KPF improves lipid metabolism and reduces hepatic steatosis

**Figure 2:**
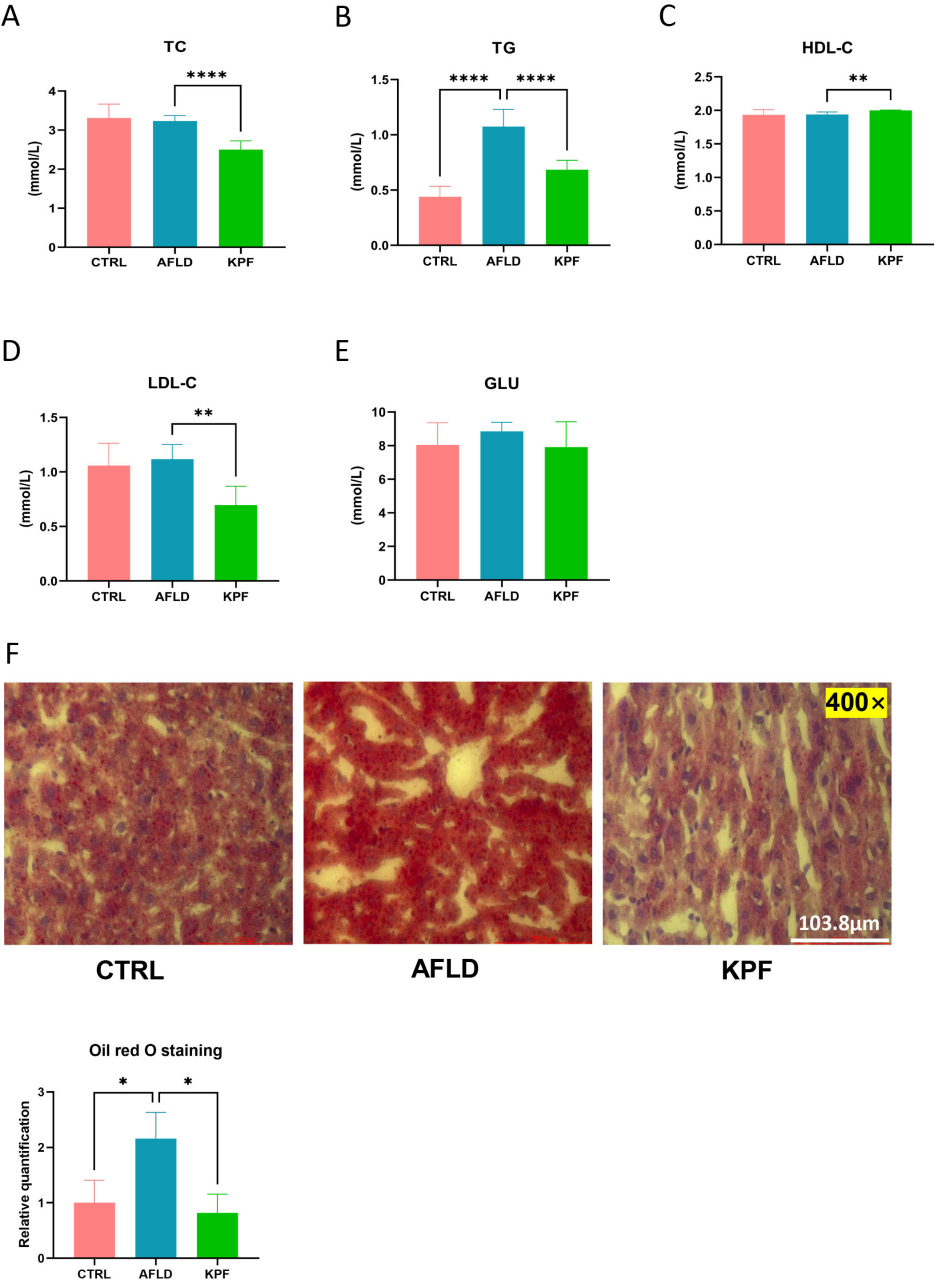
KPF Improves Lipid Metabolism and Reduces Hepatic Steatosis **(A)** Plasma total cho-lesterol (TC) levels. **(B)** Plasma triglyceride (TG) levels. **(C)** Plasma high-density lipoprotein cholesterol (HDL-C) levels. **(D)** Plasma low-density lipoprotein cholesterol (LDL-C) levels. **(E)** Plasma glucose (GLU) levels. **(F)** Representative Oil Red O-stained liver sections (magnification 400×) and quantitative bar chart of lipid droplet area. All data were analyzed by one-way ANOVA followed by Tukey’s *post-hoc* test; *p < 0.05, **p < 0.01, ****p < 0.0001 (n = 12 per group).

Lipid metabolism was assessed by measuring plasma levels of TC, TG, HDL-C, and LDL-C. KPF treatment significantly reduced TC (p < 0.0001), TG (p < 0.0001), and LDL-C levels (p < 0.01), while HDL-C levels were significantly increased compared to the AFLD group (p < 0.01) (**Figures 2A–D**). Additionally, blood glucose (GLU) levels were analyzed, and although KPF treatment tended to reduce the elevated glucose levels observed in the AFLD group, this effect did not reach statistical significance (**Figure 2E**).

Oil Red O staining of liver sections revealed extensive lipid droplet accumulation in the cytoplasm of hepatocytes in the AFLD group, indicated by numerous and larger red lipid droplets. In contrast, KPF treatment markedly reduced both the number and size of lipid droplets, demonstrating an improvement in hepatic steatosis (p < 0.05) (**Figure 2F**).

### KPF modulates Nrf2 and HO-1 expression in AFLD mice

**Figure 3:**
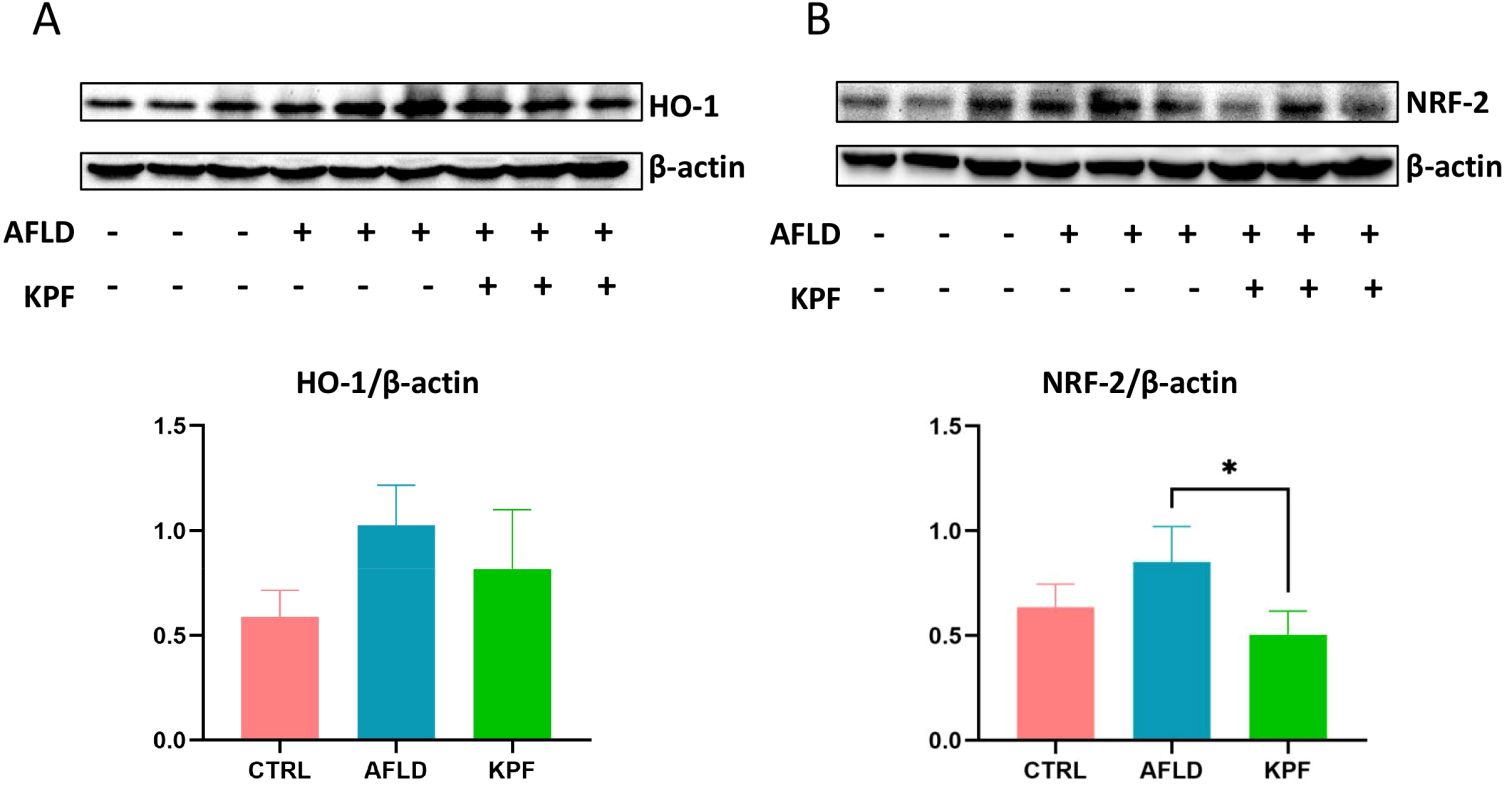
KPF Modulates Nrf2 and HO-1 Expression in AFLD Mice **(A)** Protein expression levels of HO-1 in mouse liver tissue. **(B)** Protein expression levels of Nrf2 in mouse liver tissue. KPF treatment significantly decreased Nrf2 levels and reduced HO-1 expression compared to the AFLD group. All data were analyzed by one-way ANOVA followed by Tukey’s *post-hoc* test; *p < 0.05 (n = 3 per group).

Heme oxygenase-1 (HO-1) and nuclear factor erythroid 2-related factor 2 (Nrf2) are critical regulators of antioxidant and anti-inflammatory pathways. As shown in **Figure 3**, Nrf2 protein levels were significantly elevated in the AFLD group versus the CTRL group, then dropped to a lower level after KPF treatment (p < 0.05) (**Figure 3B**). In contrast, HO-1 protein expression in the AFLD group was higher than in the CTRL group, but although it trended downward in the KPF group, this change did not reach statistical significance (**Figure 3A**). Collectively, these findings suggest that KPF reduces both Nrf2 and HO-1, although the effect is only statistically significant for Nrf2.

### KPF alters metabolite profiles and pathway enrichment in AFLD

**Figure 4:**
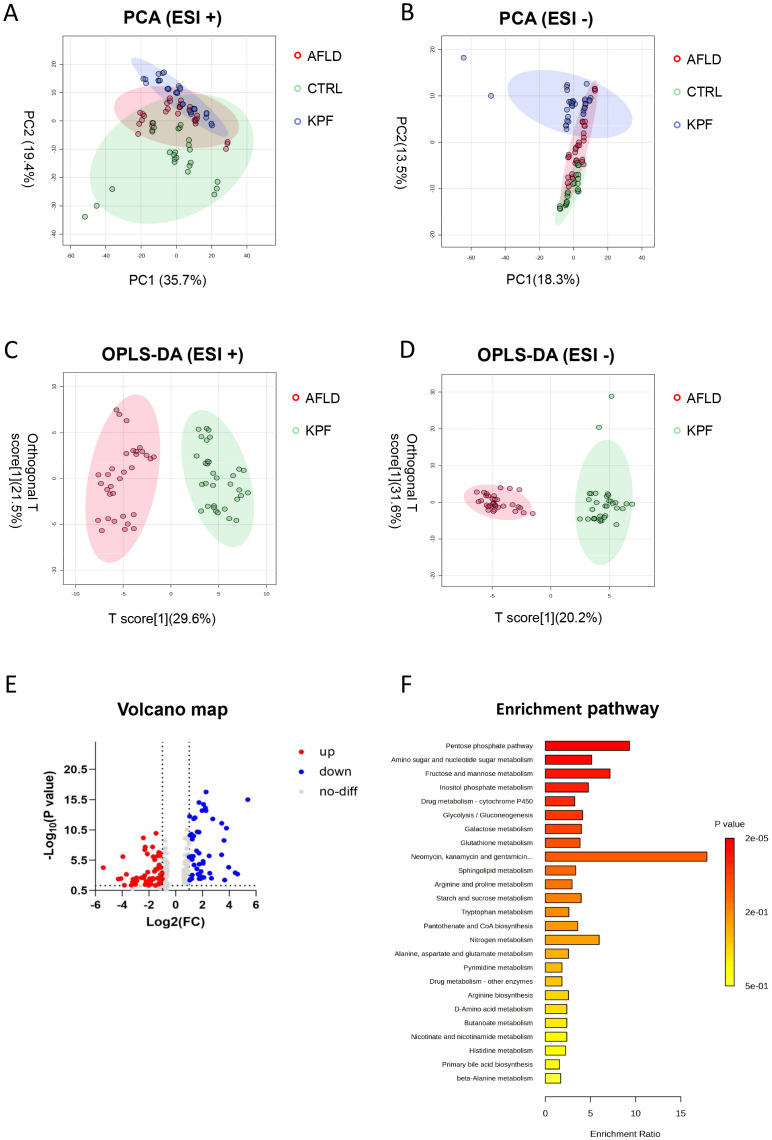
KPF Alters Metabolite Profiles and Pathway Enrichment in AFLD **(A)** PCA plot in positive ion mode (ESI+), showing separation among the CTRL, AFLD, and KPF groups. **(B)** PCA plot in neg-ative ion mode (ESI−), showing group clustering, with the KPF group trending closer to the AFLD group. **(C)** OPLS-DA plot in positive ion mode (ESI+), comparing the AFLD and KPF groups. **(D)** OPLS-DA plot in negative ion mode (ESI−), comparing the AFLD and KPF groups. **(E)** Volcano plot illustrating significantly upregulated (red) and downregulated (blue) metabolites in the KPF group compared to the AFLD group (p < 0.05, FC > 2 or FC < 0.5). **(F)** Pathway enrichment analysis of differentially expressed metabolites, highlighting metabolic pathways impacted by AFLD and modulated by KPF treatment. Data were analyzed by one-way ANOVA followed by Tukey’s *post-hoc* test, where applicable.

PCA was conducted to visualize the metabolic profiles. In positive-ion mode, the CTRL, AFLD, and KPF groups were distinctly separated, whereas in negative-ion mode, the KPF group partly overlapped with the AFLD group (**Figures 4A, B**). OPLS-DA of 213 ions between the AFLD and KPF groups (p < 0.05, FC > 2, VIP > 1) revealed clear differences in metabolite profiles (**Figures 4C, D**).

A volcano plot identified 50 significantly downregulated metabolites and 79 significantly upregulated metabolites (p < 0.05, FC > 2) in the KPF group compared to the AFLD group (**Figure 4E**). Pathway enrichment analysis of these metabolites highlighted several pathways regulated by KPF, including glycolysis/gluconeogenesis, pentose phosphate pathway, and glutathione metabolism. Multiple lipid metabolism–related pathways—sphingolipid metabolism, glyoxylate and dicarboxylate metabolism, and primary bile acid biosynthesis—were also impacted (**Figure 4F**). By matching significantly altered metabolites with endogenous metabolite databases (AUC > 0.8), we identified 32 differentially regulated endogenous metabolites (**Table 1**), which are mainly involved in oxidative stress, amino acid metabolism, and lipid metabolism, providing further mechanistic clues to KPF’s protective actions.

**Table 1:** Differential endogenous metabolites identified between AFLD and KPF groups.

NO.	m/z	Compound	KEGG ID	HMDB ID	Log_2_(FC)	-log_10_p	AUC	Retention time (mins)	Mode
1	887.5645	PI(16:0/22:4(10Z,13Z,16Z,19Z))	C00626	HMDB0009793	41.7	15.5	1.00	7.24	positive
2	834.5985	PC(18:0/22:6(4Z,7Z,10Z,13Z,16Z,19Z))	C00157	HMDB0008057	2.7	12.5	0.94	6.00	positive
3	813.6846	SM(d18:1/24:1(15Z))	C00550	HMDB0012107	10.8	11.6	1.00	6.71	positive
4	810.5981	PC(16:0/22:4(7Z,10Z,13Z,16Z))	C00157	HMDB0007988	3.3	15.0	0.97	6.13	positive
5	808.581	PC(16:0/22:5(4Z,7Z,10Z,13Z,16Z))	C00157	HMDB0007989	2.1	9.6	0.90	5.88	positive
6	794.6008	PC(20:3(5Z,8Z,11Z)/P-18:1(11Z))	C00157	HMDB0008392	2.2	9.8	0.92	5.99	positive
7	792.5873	PC(20:4(5Z,8Z,11Z,14Z)/P-18:1(11Z))	C00157	HMDB0008457	1.5	7.0	0.85	6.49	positive
8	786.5982	PC(18:1(9Z)/18:1(9Z))	C00157	HMDB0000593	4.6	14.1	0.99	6.22	positive
9	784.5814	PC(14:1(9Z)/22:2(13Z,16Z))	C00157	HMDB0007921	2.5	12.3	0.93	5.96	positive
10	772.5831	PE(14:1(9Z)/24:1(15Z))	C00350	HMDB0008882	2.3	6.1	0.85	6.09	positive
11	770.5921	PC(18:0/P-18:1(11Z))	C00157	HMDB0008062	3.4	7.9	0.89	6.78	negative
12	768.5797	PC(18:2(9Z,12Z)/P-18:1(11Z))	C00157	HMDB0008161	2.5	9.5	0.90	5.98	positive
13	766.5802	PC(18:3(6Z,9Z,12Z)/P-18:1(11Z))	C00157	HMDB0008194	2.9	7.0	0.86	5.83	positive
14	760.5811	PC(14:0/20:1(11Z))	C00157	HMDB0007879	4.2	14.7	0.99	6.13	positive
15	756.5867	PE(20:1(11Z)/P-18:1(11Z))	C00350	HMDB0009281	-1.6	3.7	0.80	5.65	positive
16	752.5355	PC(14:0/20:4(5Z,8Z,11Z,14Z))	C00157	HMDB0007883	-5.4	4.2	0.83	5.89	negative
17	750.5509	PE(20:4(5Z,8Z,11Z,14Z)/P-18:0)	C00350	HMDB0009412	-4.1	2.5	0.85	6.39	negative
18	746.5791	PE(14:0/22:1(13Z))	C00350	HMDB0008842	6.8	12.3	0.98	6.11	positive
19	744.5571	PE(14:0/22:2(13Z,16Z))	C00350	HMDB0008843	3.0	10.2	0.92	5.77	positive
20	732.5539	PC(14:0/18:1(11Z))	C00157	HMDB0007872	3.9	13.7	0.96	5.81	positive
21	730.5388	PC(14:0/18:2(9Z,12Z))	C00157	HMDB0007874	2.4	9.0	0.88	5.46	positive
22	704.5099	PE(15:0/18:1(11Z))	C00350	HMDB0008893	3.3	10.1	0.91	5.64	positive
23	703.5743	SM(d18:1/16:0)	C00550	HMDB0010169	4.8	13.7	1.00	5.53	positive
24	615.1717	Heme	C00032	HMDB0003178	3.4	6.4	0.86	5.02	negative
25	613.157	Oxidized glutathione	C00127	HMDB0003337	24.5	3.2	0.91	0.75	positive
26	506.3608	LysoPC(P-18:1(9Z)/0:0)	C04230	HMDB0010408	4.8	16.8	0.98	6.40	positive
27	407.2795	7-Ketodeoxycholic acid	C04643	HMDB0000391	2.5	6.2	0.90	1.74	positive
28	377.265	MG(0:0/20:4(5Z,8Z,11Z,14Z)/0:0)	C13856	HMDB0004666	2.5	4.5	0.82	2.00	negative
29	373.2738	Cervonoyl ethanolamide	C13828	HMDB0013627	3.4	4.8	0.84	1.31	positive
30	329.2321	9,12,13-TriHOME	C14833	HMDB0004708	-1.3	4.2	0.84	1.02	negative
31	318.3005	Phytosphingosine	C12144	HMDB0004610	12.2	8.7	0.95	0.73	positive
32	259.0224	Fructose 6-phosphate	C00085	HMDB0000124	3.7	2.4	0.81	0.83	negative

*This table summarizes 32 significantly altered endogenous metabolites identified through metabolomics analysis (AUC > 0.8). It includes m/z values, compound names, KEGG and HMDB IDs, fold change (Log2(FC)), significance (-log10(p)), AUC, retention time, and ionization mode. These metabolites are involved in key metabolic pathways, such as glycolysis/gluconeogenesis, sphingolipid metabolism, glutathione metabolism, and lipid metabolism, offering deeper insights into KPF’s protective mechanisms against AFLD.

## Discussion

The pathogenesis of AFLD entails a complex cascade, including ethanol-induced mitochondrial dysfunction, oxidative stress, inflammatory cytokine production, and lipid metabolic dysregulation (14, 15). Persistent hepatic steatosis can advance to more severe stages such as alcoholic steatohepatitis (ASH), fibrosis, or hepatocellular carcinoma (HCC) (16). These challenges reinforce the urgent need for novel therapeutic agents to halt AFLD progression.

In this study, we demonstrated that kaempferol (KPF) ameliorates ethanol-induced hepatic damage in an AFLD mouse model. KPF significantly lowered key indicators of liver injury (ALT, AST), while concurrently sustaining body weight. Histological analysis corroborated these biochemical findings, revealing reduced steatosis and restored normal liver architecture. These results echo previous reports on the hepatoprotective attributes of KPF (17, 18) highlighting its capacity to reverse alcohol-driven structural damage.

Oxidative stress is central to AFLD progression, as excessive ROS can overwhelm hepatic antioxidant defense (15, 19, 20). Here, KPF counteracted oxidative stress by diminishing MDA levels—an index of lipid peroxidation—and boosting SOD and GSH-Px. This aligns with prior evidence that KPF lessens ROS production and rebuilds redox balance (21). Interestingly, both Nrf2 and HO-1 were elevated in the AFLD group, implying that Nrf2/HO-1 activation may act as a compensatory response under severe oxidative stress (22–24). After KPF intervention, Nrf2 and HO-1 both declined compared with the AFLD group. While this may initially appear counterintuitive, it suggests that reduced overall oxidative pressure by KPF lessens the requirement for high-level Nrf2/HO-1 activation.

Moreover, KPF’s effects on lipid metabolism are substantial. It lowered plasma TC, TG, and LDL-C while raising HDL-C, underscoring its broad regulatory role in lipid handling. Oil Red O staining further confirmed reduced hepatic lipid droplet formation. Compared with monotherapy approaches, KPF’s multi-target strategy—encompassing antioxidant and lipid-lowering actions—could offer a more effective blueprint for AFLD management, especially given the multifactorial nature of alcohol-induced liver injury.

Mechanistically, the metabolomic data reinforce the notion that KPF exerts a coordinated influence on multiple metabolic pathways. We found KPF to affect glycolysis/gluconeogenesis and the pentose phosphate pathway, critical sources of NADPH for antioxidant defense (25–29). It also influenced glutathione metabolism, cytochrome P450 function, and beta-alanine metabolism, processes tied to ROS detoxification and hepatic homeostasis (30–32). Alterations in sphingolipid metabolism and primary bile acid biosynthesis further highlight KPF’s role in modulating lipid-associated pathways (33, 34). Notably, we identified 32 endogenous metabolites, including phospholipids, sphingolipids, and oxidative stress–linked compounds (oxidized glutathione, heme), suggesting a multi-pronged mechanism of action rather than a single linear route. Although we did not directly measure hepatic expression of lipid metabolism-related transcription factors such as SREBP-1c or PPARα in this study, investigating these regulatory pathways via RT-qPCR analyses will be essential in future research to fully elucidate KPF’s hypolipidemic mechanisms.

These comprehensive findings stress that KPF’s protective benefits likely stem from modulating oxidative stress, fine-tuning lipid metabolism, and rebalancing key metabolic pathways. Such multi-level intervention could be especially valuable given the complex etiology of AFLD, where multiple signaling cascades converge to drive disease progression.

## Study Limitations

While this work provides important insights, several limitations warrant consideration. First, the relatively short experimental duration constrains our understanding of KPF’s long-term efficacy and safety. Second, the animal model may not fully replicate the chronic and progressive nature of human AFLD. Third, we only tested a single KPF dose, leaving the dose-response relationship uncharacterized. Fourth, although we measured key oxidative stress markers (e.g., SOD, GSH-Px, MDA), catalase activity was not assessed and should be evaluated in future experiments. Fifth, Nrf2 protein expression was measured only in total liver lysates; future studies should specifically assess Nrf2 nuclear translocation to accurately evaluate its activation status. Finally, metabolomics was performed exclusively on plasma, chosen for its straightforward sample preparation and established detection methods. Future studies would benefit from integrating liver tissue metabolomics to elucidate hepatic-specific metabolic pathways and to further validate the biological relevance and clinical applicability of these findings.

## Conclusion

This study positions kaempferol (KPF) as a promising dietary-derived therapeutic for AFLD, demonstrating efficacy in mitigating liver injury, decreasing oxidative stress, and regulating lipid metabolism via the Nrf2/HO-1 axis. Metabolomic analysis revealed multiple metabolic pathways potentially modulated by KPF, including glycolysis/gluconeogenesis, sphingolipid metabolism, and glutathione metabolism, thereby underscoring its multi-targeted mode of action. Although further validation is needed to confirm whether these metabolite changes translate into reliable clinical markers, our findings establish a robust foundation for the development of KPF-based interventions. Overall, this study supports the potential of food-derived KPF as a functional strategy for preventing and managing AFLD.

## Data Availability

The original contributions presented in this study are included in the article/**Supplementary Materials**. Further inquiries can be directed to the corresponding author.

## Supplementary material

Supplementary Table 1 abundance of positive mode ions.

Supplementary Table 2 abundance of negative mode ions.
